# Identification of a novel heterozygous guanosine monophosphate reductase (*GMPR*) variant in a patient with a late‐onset disorder of mitochondrial DNA maintenance

**DOI:** 10.1111/cge.13652

**Published:** 2019-11-14

**Authors:** Ewen W. Sommerville, Ilaria Dalla Rosa, Masha M. Rosenberg, Francesco Bruni, Kyle Thompson, Mariana Rocha, Emma L. Blakely, Langping He, Gavin Falkous, Andrew M. Schaefer, Patrick Yu‐Wai‐Man, Patrick F. Chinnery, Lizbeth Hedstrom, Antonella Spinazzola, Robert W. Taylor, Gráinne S. Gorman

**Affiliations:** ^1^ Wellcome Centre for Mitochondrial Research, Institute of Neuroscience Newcastle University Newcastle upon Tyne UK; ^2^ Department of Clinical and Movement Neurosciences, UCL Queens Square Institute of Neurology, Royal Free Campus University College London London UK; ^3^ Department of Biology Brandeis University Waltham MA; ^4^ Department of Biosciences, Biotechnologies and Biopharmaceutics University of Bari “ldo Moro” Bari Italy; ^5^ NIHR Biomedical Research Centre at Moorfields Eye Hospital and UCL Institute of Ophthalmology London UK; ^6^ MRC Mitochondrial Biology Unit University of Cambridge Cambridge UK; ^7^ Cambridge Centre for Brain Repair, Department of Clinical Neurosciences University of Cambridge Cambridge UK; ^8^ Department of Clinical Neuroscience & Medical Research Council Mitochondrial Biology Unit School of Clinical Medicine, University of Cambridge Cambridge UK; ^9^ Department of Chemistry Brandeis University, 415 South St. Waltham MA; ^10^ MRC Centre for Neuromuscular Diseases UCL Institute of Neurology and National Hospital for Neurology and Neurosurgery London UK

**Keywords:** GMPR, mitochondrial DNA maintenance, multiple mtDNA deletions, PEO, whole exome sequencing

## Abstract

Autosomal dominant progressive external ophthalmoplegia (adPEO) is a late‐onset, Mendelian mitochondrial disorder characterised by paresis of the extraocular muscles, ptosis, and skeletal‐muscle restricted multiple mitochondrial DNA (mtDNA) deletions. Although dominantly inherited, pathogenic variants in *POLG*, *TWNK* and *RRM2B* are among the most common genetic defects of adPEO, identification of novel candidate genes and the underlying pathomechanisms remains challenging. We report the clinical, genetic and molecular investigations of a patient who presented in the seventh decade of life with PEO. Oxidative histochemistry revealed cytochrome *c* oxidase‐deficient fibres and occasional ragged red fibres showing subsarcolemmal mitochondrial accumulation in skeletal muscle, while molecular studies identified the presence of multiple mtDNA deletions. Negative candidate screening of known nuclear genes associated with PEO prompted diagnostic exome sequencing, leading to the prioritisation of a novel heterozygous c.547G>C variant in *GMPR* (NM_006877.3) encoding guanosine monophosphate reductase, a cytosolic enzyme required for maintaining the cellular balance of adenine and guanine nucleotides. We show that the novel c.547G>C variant causes aberrant splicing, decreased GMPR protein levels in patient skeletal muscle, proliferating and quiescent cells, and is associated with subtle changes in nucleotide homeostasis protein levels and evidence of disturbed mtDNA maintenance in skeletal muscle. Despite confirmation of GMPR deficiency, demonstrating marked defects of mtDNA replication or nucleotide homeostasis in patient cells proved challenging. Our study proposes that *GMPR* is the 19th locus for PEO and highlights the complexities of uncovering disease mechanisms in late‐onset PEO phenotypes.

## Graphical abstract

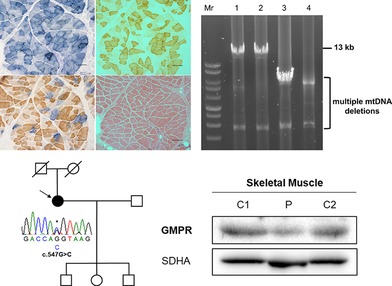

## 1 INTRODUCTION

Autosomal dominant progressive external ophthalmoplegia (adPEO) is a mitochondrial DNA (mtDNA) maintenance disorder characterised by restriction of the extraocular muscles, ptosis, and secondary, clonally expanded skeletal muscle‐restricted multiple mtDNA deletions.^1^ The spectrum of PEO clinical phenotypes are broad, ranging from isolated PEO to fatal, multisystem PEO‐plus disorders,^2^ while pathogenic, dominantly inherited variants of *POLG* (MIM 174763), *TWNK* (MIM 606075) and *RRM2B* (MIM 604712) are among the most common causes of adulthood to late‐onset PEO.^3^ Although next‐generation DNA sequencing technologies have enhanced its genetic diagnosis and led to the identification of novel genes, PEO candidate variant prioritisation is highly challenging due to mild phenotypic expression at a subcellular level.

Maintaining a balance of all four deoxyribonucleotides (dNTPs), the building blocks for DNA synthesis, is essential for mtDNA replication and is intricately regulated through synthesis and degradation.^4^ In proliferating (dividing) cells, dNTPs for mtDNA are predominantly synthesised de novo in the cytosol through ribonucleotide reduction by ribonucleotide reductase (RNR), which is composed of the large R1 and small R2 subunits.^5^ A small proportion of dNTPs are also derived from recycling via the cytosolic and mitochondrial deoxyribonucleotide salvage pathways. In quiescent (non‐dividing) cells, nuclear DNA replication is suspended. As a consequence, cytosolic de novo synthesis is strongly reduced. DNA replication in mitochondria continues, instead relying upon the rate‐limiting enzymes thymidine kinase 2 (TK2) and deoxyguanosine kinase (DGUOK), via the mitochondrial deoxyribonucleotide salvage pathway^6^ and limited cytosolic de novo synthesis through the alternative RNR containing the p53R2 subunit.^7^, ^8^, ^9^, ^10^

Pathogenic variants in *ABAT* (MIM 137150), *DGUOK* (MIM 601465), *MPV17* (MIM 137960), *RRM2B*, *SUCLA2* (MIM 603921), *SUCLG1* (MIM 611224), *TK2* (MIM 188250) and *TYMP* (MIM 131222), encoding proteins involved in dNTP homeostasis, are known to cause quantitative (depletion) or qualitative (multiple deletions) disorders of mtDNA maintenance.^11^, ^12^, ^13^, ^14^, ^15^, ^16^, ^17^, ^18^, ^19^, ^20^, ^21^ Of these, *RRM2B* and *TYMP* encode cytosolic enzymes, which support an assumption that there is mixing of cytosolic and mitochondrial dNTP pools. This is further supported by the identification of pyrimidine nucleotide carriers 1 and 2 (PNC1/2) and equilibrative nucleotide transporter 1 (ENT1), which exchange pyrimidine or purine nucleotides from the cytosol through the mitochondrial membrane to the matrix.^22^, ^23^, ^24^, ^25^ Therefore, a cytosolic nucleotide metabolism defect can negatively influence DNA replication in mitochondria and lead to defects in mtDNA maintenance.

Here, we present a patient with a novel heterozygous c.547G>C variant in *GMPR* (MIM 139265) encoding the cytosolic purine metabolism enzyme guanosine monophosphate reductase with late onset PEO. Our functional data corroborated the mild phenotypic expression leading us to propose that *GMPR* is a rare, novel candidate locus for adulthood‐onset adPEO with disordered mtDNA maintenance.

## 2 METHODS

### 2.1 Histopathology, biochemical and molecular genetic studies

Skeletal muscle biopsy was subjected to haemotoxylin and Eosin (H&E) staining, cytochrome *c* oxidase (COX), succinate dehydrogenase (SDH), sequential COX‐SDH histochemical reactions and quadruple immunofluorescence assay analysis.^26^ Whole mitochondrial genome sequencing was performed to exclude pathogenic variants. The presence of mtDNA rearrangements and mtDNA copy number was assessed using established diagnostic long‐range PCR^27^ and real‐time PCR assays.^28^

### 2.2 Whole exome sequencing, candidate variant prioritisation and genetic studies

Exome capture of fragmented patient blood genomic DNA was attained and WES was performed as previously described.^29^ Details of WES analysis, candidate variant prioritisation, Sanger sequencing and cDNA studies in skeletal muscle and cultured fibroblasts are outlined in Supporting Information.

### 2.3 In vitro studies

In vitro experiments using hGMPR2 and hGMPR2‐G183R mutant were performed as previously described.^30^, ^31^ Details of cloning, mutagenesis, overexpression and purification are outlined in Supporting Information.

### 2.4 Cell culture

Cells were incubated at 37°C with 5% CO_2_. Cultured patient and control fibroblasts were grown in minimum essential medium (MEM) with 10% foetal bovine serum, 1% MEM vitamins, 1% non‐essential amino acids, 50 U ml^−1^ penicillin, 50 μg ml^−1^ streptomycin, 1 mM sodium pyruvate solution, 25 mg ml^−1^ uridine solution and 2 mM l‐glutamine.

Quiescent (non‐dividing) cells were generated to repress cytosolic de novo nucleotide synthesis and limit the supply of dNTPs to mitochondria. Once cultured fibroblast cell lines were confluent, the culture medium was replaced with MEM containing 0.1% dialysed foetal bovine serum. Cells were incubated with quiescent medium for 10‐14 days, with medium replenished every 2‐3 days. Guanosine (G) was supplemented in quiescent medium at a final concentration of 100 μM for the whole duration of the serum starvation period.

### 2.5 Cellular and sub‐mitochondrial fractionation

HeLa and HEK293T cells were fractionated as described previously,^32^ with few modifications (Supporting Information).

### 2.6 Immunoblotting

Cell lysates (50 μg) and skeletal muscle homogenate (25‐50 μg) were separated by 10% or 12% SDS‐PAGE and electrophoretically transferred to polyvinylidene difluoride (PVDF) membranes (Bio‐Rad). Primary antibodies are listed in Table SS1. Both GMPR primary antibodies (ab118751, Abcam; SAB1101144, Sigma) were specific to GMPR but not GMPR2.

For colocalisation studies, 30 μg of protein from each fraction were analysed by immunoblotting using primary antibodies as follows: GMPR (ab118751, Abcam), GDH (custom made), NDUFB8 (as described above), eIF4E (9742, Cell Signaling Technology), AIF (4642, Cell Signaling Technology) and EF‐Tu (custom made).

Following incubation with horseradish peroxidase‐conjugated secondary antibodies (Dako) for 1 hour at room temperature, detected proteins were visualised with Clarity Western ECL substrate (Bio‐Rad) or Amersham ECL Prime Western Blotting Detection Reagent (GE Healthcare, Life Sciences), and signals were visualised using the Bio‐Rad ChemiDoc MP with Image Lab software according to manufacturer's guidelines.

### 2.7 Confocal microscopy and nucleoid organisation

Confocal microscopy of proliferating and quiescent cells was performed as previously described.^33^, ^34^ Further details and assessment of nucleoid organisation in skeletal muscle are outlined in Supporting Information.

### 2.8 In vitro mitochondrial protein synthesis

Newly synthesised mitochondrial proteins were assessed as described previously.^35^

### 2.9 Cytosolic and mitochondrial dNTP pool determination

Approximately 1.5 × 10^8^ fibroblasts under quiescent conditions were homogenised in 210 mM mannitol, 70 mM sucrose, 10 mM Tris HCl, pH 7.5, 0.2 mM EGTA, and 0.5 mg ml^−1^ BSA using a 23‐gauge needle (total fraction). Homogenates were then centrifuged at 1000*g* for 5 minutes, and the crude mitochondrial fraction in the supernatant was pelleted at 19000 g for 20 minutes. dNTPs were extracted from total homogenates or mitochondrial pellets and dNTP pools were measured by the polymerase‐based method as described previously.^36^

### 2.10 Quantitative and qualitative assessments of mtDNA in proliferating and quiescent cells

DNA was extracted using DNeasy Blood & Tissue Kit (Qiagen), according to the manufacturer instructions. Quantification of relative mtDNA copy number in proliferating and quiescent cells was performed as described previously.^36^ Long‐range PCR was performed as described previously,^27^ with increasing concentrations of DNA template.

## 3 RESULTS

### 3.1 Case report

**Figure 1:**
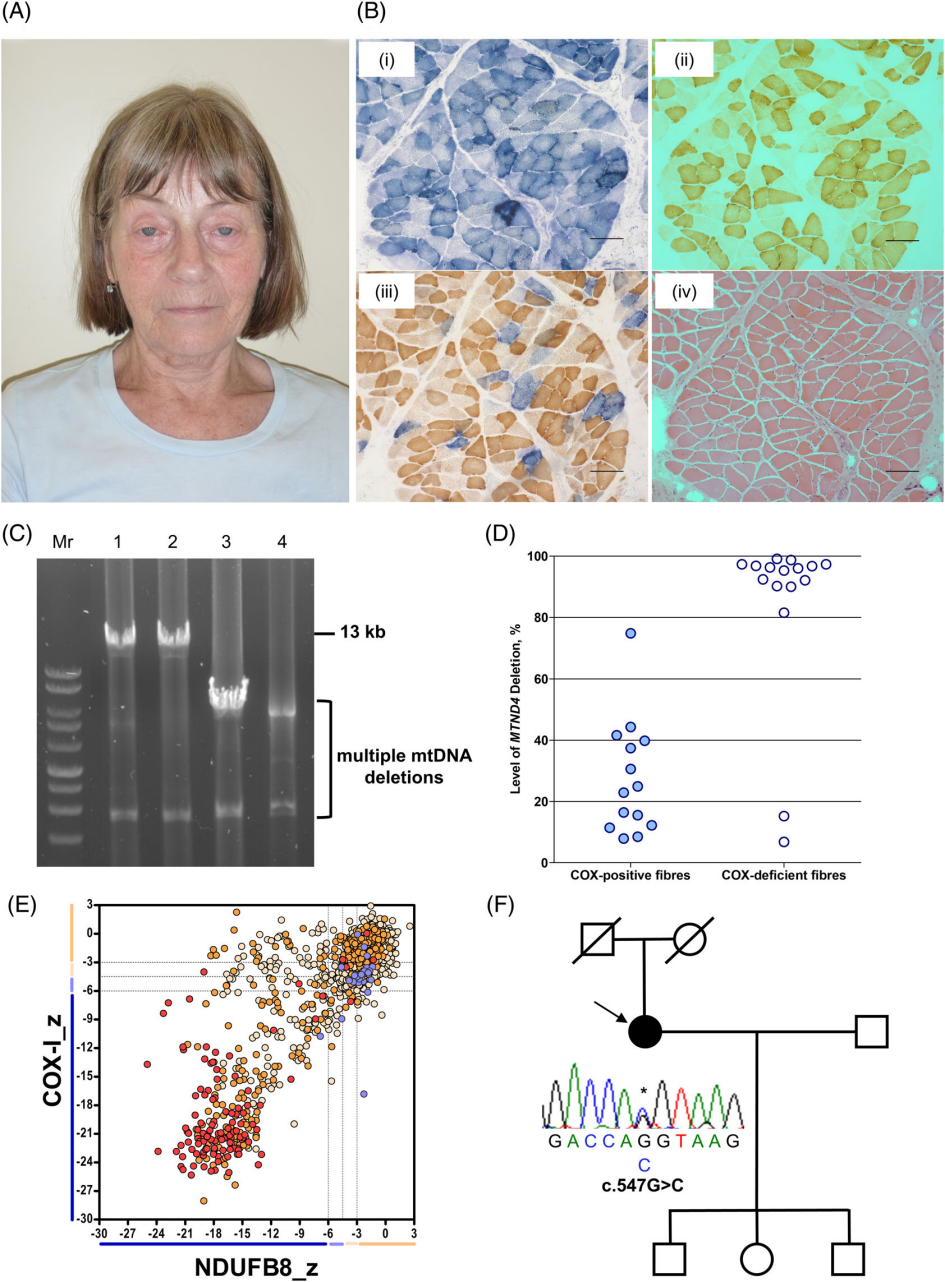
Clinical, histopathologic and molecular characterisation of a patient harbouring a novel heterozygous c.547G>C *GMPR* variant. A, Ophthalmological features of the patient with PEO harbouring a novel heterozygous *GMPR* variant, highlighting bilateral ptosis and frontalis muscle hyperactivity. B, A skeletal muscle biopsy from the patient was subjected to (a) COX, (b) SDH, (c) sequential COX‐SDH histochemical reactions and (d) haemotoxylin and eosin (H&E) staining. Scale bar represents 100 μM. C, 13‐kb long‐range PCR assay of skeletal muscle mtDNA demonstrating multiple mtDNA deletions in the patient (lane 4) compared with aged‐matched controls (lanes 1 and 2) and a patient with a single, large‐scale mtDNA deletion (lane 3). D, Quantitative single‐fibre real‐time PCR assay reveals that the majority of COX‐deficient fibres exhibit clonally expanded multiple mtDNA deletions involving the *MTND4* gene. E, Mitochondrial respiratory chain expression profile showing NDUFB8 (complex I), COX‐I (complex IV) and porin levels in individual patient skeletal muscle fibres. Each dot represents an individual muscle fibre, colour coded according to mitochondrial mass (very low, blue; low, light blue; normal, light orange; high, orange; and very high, red). Black dashed lines represent the SD limits for the classification of fibres. Lines adjacent to the *X*‐ and *Y*‐axis represent the levels of NDUFB8 and COX‐I (beige, normal; light beige, intermediate (+); light blue, intermediate (−); and blue, negative]. F, Family pedigree and Sanger sequencing confirmation of the novel c.547G>C *GMPR* variant in the index case [Colour figure can be viewed at http://wileyonlinelibrary.com]

The patient is a 73‐year‐old female, the only child born to non‐consanguineous parents, who presented with a history of late adulthood‐onset PEO. At 60 years old, she underwent corrective strabismus surgery but complained of diplopia post‐surgery. She also reported mild bilateral ptosis at 69 years old. Upon clinical examination she had marked PEO with subtle asymmetry, mild asymmetric ptosis (right>left), exotropia of the right eye and mild orbicularis oculi weakness (Figure 1A). Mild facial and proximal muscle weakness (MRC 4+/5) were also noted. Brain MRI was normal but there was no comment on the extraocular muscles. There was no reported family history of any eye movement or neuromuscular disorder. She has three asymptomatic children.

### 3.2 A skeletal muscle biopsy from the patient displays hallmarks of a mtDNA maintenance disorder

A muscle biopsy revealed approximately 15% cytochrome *c* oxidase (COX)‐deficient fibres and occasional ragged‐red fibres demonstrating subsarcolemmal mitochondrial accumulation (Figure 1B). Long‐range PCR of skeletal muscle DNA revealed multiple mtDNA deletions (Figure 1C), confirmed by quantitative single‐fibre real‐time PCR in which the majority of COX‐deficient fibres were shown to harbour clonally expanded mtDNA rearrangements involving the *MTND4* gene (Figure 1D). Quadruple OXPHOS immunofluorescence assay of individual muscle fibres showed a greater proportion of fibres with reduced NDUFB8 (complex I) levels and a population of fibres with equal loss of NDUFB8 and MT‐COI (complex IV) proteins (Figure 1E), suggestive of a mtDNA maintenance disorder.^26^

### 3.3 Identification of a novel heterozygous c.547G>C *GMPR* variant

Candidate screening of nuclear genes associated with adulthood‐onset PEO and multiple mtDNA deletions (*POLG*, NM_002693.2; *RRM2B*, NM_015713.4; *SLC25A4*, NM_001151.4; *TWNK*, NM_021830.5; *POLG2*, NM_007215.4; *TK2*, NM_004614.4; *RNASEH1*, NM_002936.5) failed to detect pathogenic or likely pathogenic variants, prompting whole exome sequencing.

We implemented a custom‐variant filtering strategy that anticipated either a dominant or recessive aetiology, prioritising rare or novel variants in nuclear genes encoding DNA replisome machinery or mitochondrial‐localised proteins.^37^ Assessment of rare or novel variants in known nuclear genes associated with disordered mtDNA maintenance, including genes screened diagnostically, did not reveal pathogenic or likely pathogenic variants. Our analysis identified a novel heterozygous c.547G>C variant in exon 5 of the *GMPR* (NM_006877.3) gene encoding guanosine monophosphate reductase. GMPR catalyses the NADPH‐dependent deamination of the ribonucleotide guanosine monophosphate (GMP) to inosine monophosphate (IMP) in the cytosol, thereby maintaining purine nucleotide pools.^38^ The novel variant was absent from 378 in‐house controls and GnomAD (http://gnomad.broadinstitute.org/). Sanger sequencing confirmed the variant, but unfortunately familial segregation studies were not possible (Figure 1F). Analysis of copy number variants (CNVs) identified by WES did not disclose rearrangements encompassing *GMPR*. A single nucleotide polymorphism array was also negative for additional rare variants in *GMPR* or rearrangements. We also submitted *GMPR* to the GeneMatcher initiative (https://genematcher.org/ ), however this failed to deliver matching submissions.

In the absence of other candidate variants and the known function of GMPR, we prioritised further studies of the novel *GMPR* variant.

### 3.4 The novel c.547G>C variant causes aberrant splicing and GMPR depletion

**Figure 2:**
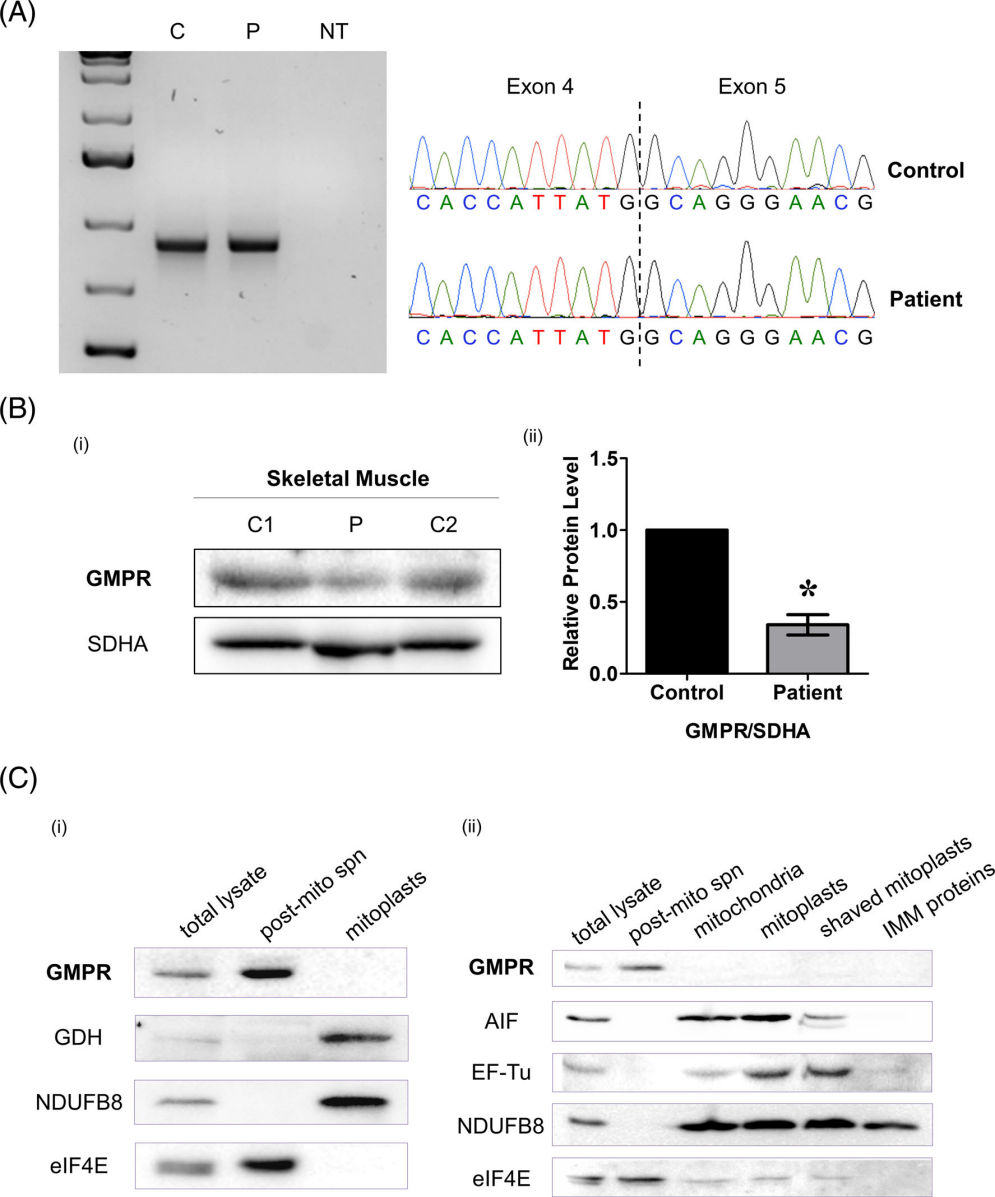
Genetic characterisation of the novel *GMPR* variant in skeletal muscle. A, Amplification of control and patient skeletal muscle‐derived cDNA across *GMPR* exons 3‐7 and sequencing chromatograms showing wild‐type PCR products from control and patient skeletal muscle. NT, No template. B, Steady‐state GMPR protein levels in control and patient skeletal muscle homogenate. SDHA was used as a loading control. C, Subfractionation of (a) HEK293T and (b) HeLa cells subjected to immunoblotting with a GMPR antibody and markers for each subfraction: GDH and EF‐Tu—mitochondrial matrix; AIF—mitochondrial inner membrane space; NDUFB8—mitochondrial inner membrane; eIF4E—cytosol. All subfractions were prepared from the same HeLa or HEK293T lysate [Colour figure can be viewed at http://wileyonlinelibrary.com]

The c.547G>C *GMPR* variant was initially predicted to cause a p.Gly183Arg missense change. Our in vitro data assessing a human GMPR2‐G183R mutant confirmed that the Gly183 residue is critical for enzymatic activity (Figure SS3) as it is close to the active site.^39^ However, further experiments of the c.547G>C variant, occurring at the last nucleotide of exon 5, showed that it led to aberrant splicing of the GMPR transcript. We assessed splicing by sequencing skeletal muscle‐derived cDNA from the patient and an age‐matched control. Following RT‐PCR, we observed only wild‐type products corresponding to full‐length *GMPR* cDNA from control and patient skeletal muscle (Figure 2A). Next, we assessed splicing by sequencing fibroblast‐derived cDNA from patient and control fibroblasts. Only wild‐type PCR products were observed in patient and control fibroblasts without emetine treatment (Figure SS1A). However, a short, abnormal PCR product was observed in patient cells after 10‐hour emetine treatment to inhibit nonsense mediated decay. Sequencing of the abnormal PCR product revealed a secondary trace with skipping of exon 5, removing nucleotides r.466 to r.547 (r.466_547del) from the transcript (Figure SS1B). The predicted effect at the protein level was a frameshift deletion, p.Ala156Valfs*17.

Immunoblotting revealed that steady‐state GMPR protein levels were decreased to approximately 34% in patient skeletal muscle homogenate, which is relative to age‐matched controls (Figure 2B). GMPR protein levels were also decreased in proliferating (dividing) and quiescent (non‐dividing) patient and control cell lysates (Figure SS2B). These data were consistent with nonsense‐mediated decay of the mutant transcript. Interestingly, GMPR protein levels were reduced in both control and patient cells in quiescent state, suggesting that GMPR activity is reduced in cells that have exited the cell‐cycle.

### 3.5 Patient skeletal muscle and fibroblasts do not show marked OXPHOS defects

Core OXPHOS subunit protein levels were unaffected in skeletal muscle homogenate (Figure SS2A), demonstrating that the reduced complex I and IV subunit levels in individual muscle fibres observed by quadruple immunofluorescence assay were not necessarily readily detectable at a tissue homogenate level. OXPHOS subunit protein levels were also unaffected in proliferating and quiescent patient cell, relative to control cells (Figure S2B).

### 3.6 GMPR is a cytosolic purine metabolism enzyme

GMPR is not a known mitochondrial protein and is not listed in the MitoCarta compendium.^40^ With the exception of PSort, all localisation algorithms (TargetP, MitoProt II, Predotar, and MitoFates) predicted a low probability of mitochondrial localisation and no targeting pre‐sequence. We performed subfractionation and immunoblotting of HEK293T and HeLa cells to experimentally confirm GMPR localisation. Immunoblotting of whole cell and mitochondrial‐free lysates using markers for the mitochondrial matrix (GDH, EF‐Tu), intermembrane space (AIF), inner membrane (NDUFB8) and cytosol (eIF4E) showed that GMPR does not localise to mitochondria and is present only in the cytosol (Figure 2C).

### 3.7 Subtle alterations in nucleotide homeostasis protein levels and mtDNA maintenance markers in patient skeletal muscle

**Figure 3:**
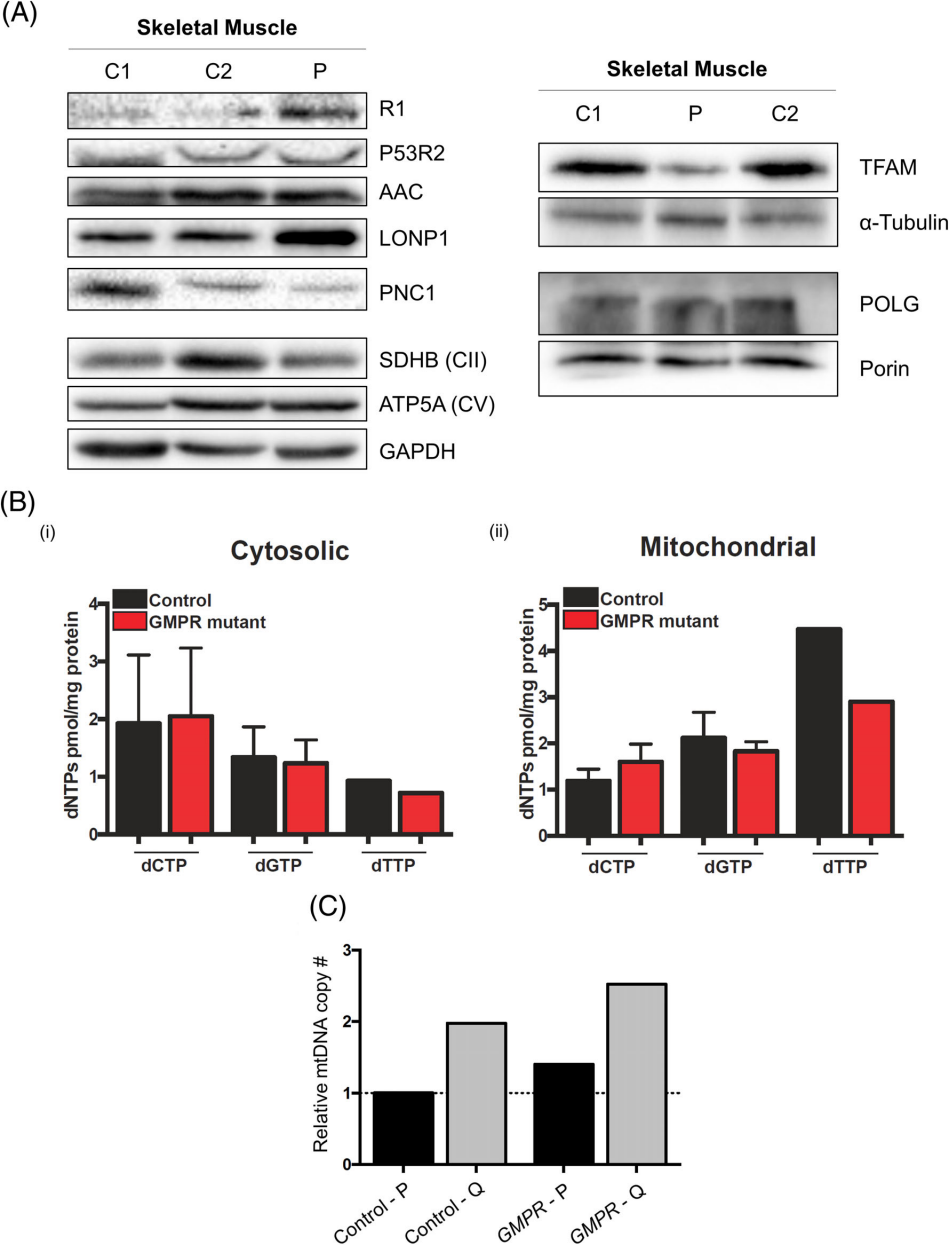
Assessment of nucleotide homeostasis and mtDNA machinery markers in skeletal muscle and cytosolic and mitochondrial dNTP measurements in quiescent cells. A, Steady‐state proteins levels of nucleotide homeostasis and mtDNA maintenance markers in control and *GMPR* patient skeletal muscle homogenates. GAPDH, α‐tubulin and porin were used as loading controls. OXPHOS subunits SDHB (CII) and ATP5A (CV) were also used as markers to confirm protein loading. B, Cytosolic (left) and mitochondrial (right) dNTP levels in quiescent GMPR mutant and control fibroblasts. C, Relative mtDNA copy number in *GMPR* patient and control proliferating (P) and quiescent (Q) fibroblasts. Relative mtDNA copy number was expressed as fold change relative to one proliferating control [Colour figure can be viewed at http://wileyonlinelibrary.com]

We postulated that GMPR deficiency would increase the levels of its substrate GMP, leading to increased GTP levels and therefore altering dNTP pools. Hence, this could perturb the purine balance and, in turn, affect the de novo synthesis of dNTPs. We assessed protein levels of the RNR subunits, R1 and p53R2, and nucleotide transporters PNC1 and AAC1 (Figure 3A, left panel). Steady‐state levels of the large R1 subunit of the RNR were mildly increased, whereas levels of the small p53R2 subunit and AAC1, the mitochondrial ADP/ATP carrier, were unaffected. By contrast, PNC1, a mitochondrial pyrimidine nucleotide carrier, was decreased in patient skeletal muscle.

mtDNA levels were at the high range of normal (data not shown) whereas the level of mtDNA packaging protein transcription factor A (TFAM) was markedly decreased in *GMPR* patient skeletal muscle (Figure 3A, right panel) and LONP1, which degrades TFAM not bound to mtDNA, was elevated. Levels of the mitochondrial DNA polymerase gamma large subunit (POLG) were unchanged.

### 3.8 Cytosolic and mitochondrial dNTP levels in quiescent *GMPR* patient cells are unaltered

To further test our hypothesis, we quantified cytosolic and mitochondrial dNTPs (dCTP, dGTP, and dTTP) in quiescent patient and control cells. dATPs were not measurable with our PCR‐based method. However, there were no changes in either cytosolic or mitochondrial dNTP levels, relative to controls (Figure 3B). These results indicate that dNTP pools are unaffected in the in vitro cultured patient cells. According to this, mtDNA maintenance was not altered, as mtDNA copy number was also normal in proliferating and quiescent patient cells (Figure 3C) and deletions were undetectable (see the next section).

To investigate whether the possible presence of mtDNA deletions in patient cells could alter mitochondrial protein synthesis, we assessed de novo synthesis of mtDNA‐encoded OXPHOS subunits in proliferating and quiescent cells by ^35^S‐methionine labelling. This showed a slight decrease of nascent mitochondrial proteins in quiescent patient cells, while proliferating cells were unaffected (Figure SS2C).

### 3.9 Mitochondrial networks and nucleoid morphology in proliferating and quiescent patient cells

**Figure 4:**
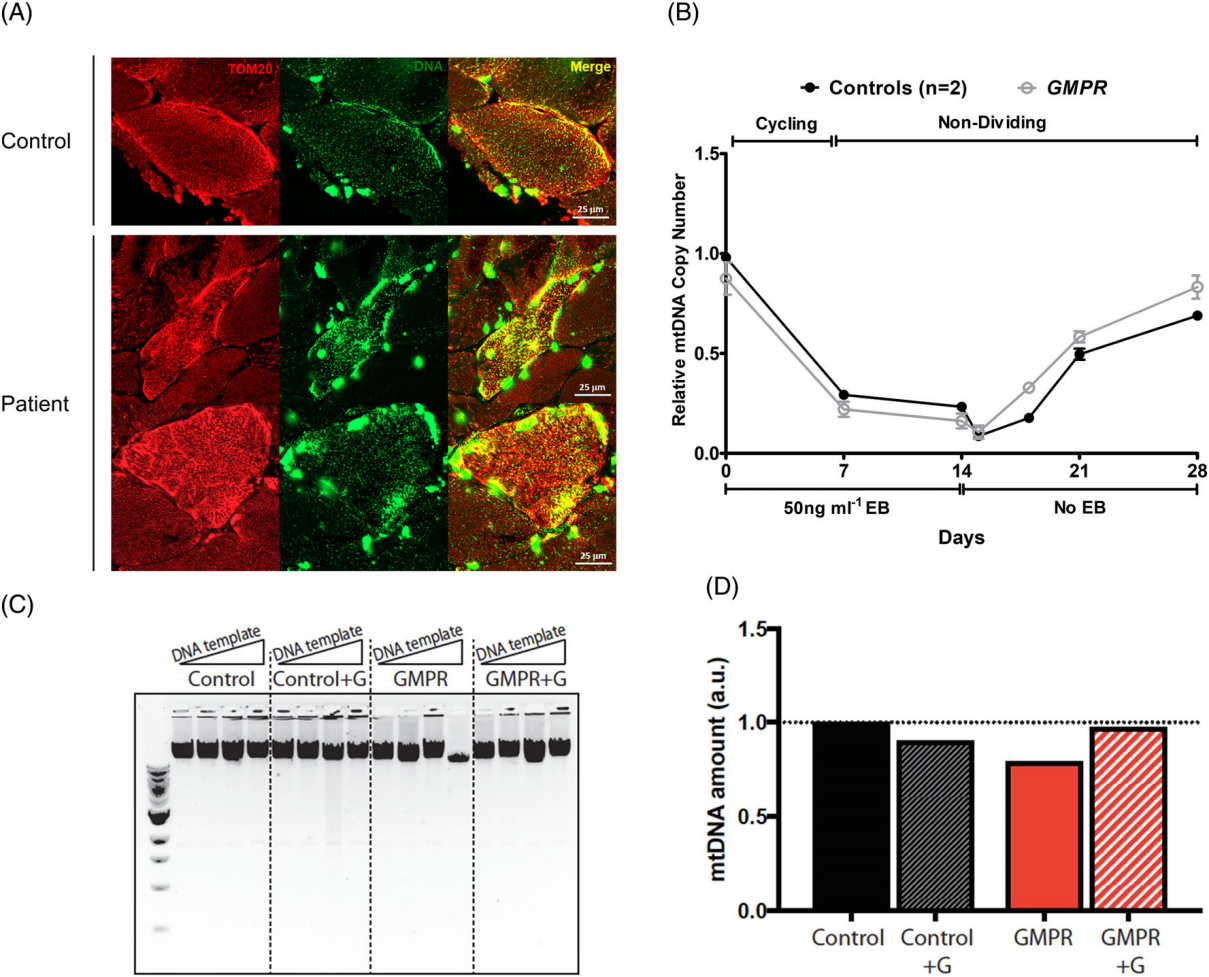
Nucleoid and mitochondrial network morphology in skeletal muscle, mtDNA depletion‐repletion studies and guanosine supplementation in non‐dividing cells. A, Confocal images of patient and control muscle sections labelled with antibodies to the mitochondrial membrane protein TOM20 (red) and DNA (green). B, Non‐dividing patient and control cells were depleted of mtDNA using the intercalating agent ethidium bromide for 14 days. Ethidium bromide was then removed and mtDNA replenishment was followed for 14 days. Relative mtDNA copy number was expressed as fold change relative to one control. Student's *t* test was performed for statistical comparison between control and patient cell mtDNA copy number. C,D, Patient and control fibroblasts were put into quiescence by serum starvation for 14 days with or without guanosine supplementation. mtDNA copy number (C) and mtDNA deletions (D) were assessed at the end of the treatment. Increasing amounts of DNA (10, 20, 40 and 80 μg) were used as template for long‐range PCR [Colour figure can be viewed at http://wileyonlinelibrary.com]

To investigate mtDNA organisation, we fixed and labelled sections of control and *GMPR* patient skeletal muscle with anti‐TOM20 and anti‐DNA antibodies. Patient skeletal muscle fibres were characterised by an increased number of enlarged nucleoids, compared with an age‐matched control (Figure 4A). Furthermore, there was increased TOM20 staining in patient fibres that were suggestive of mitochondrial biogenesis. Hence, enlarged nucleoids in patient skeletal muscle fibres could be secondary to increased mitochondrial biogenesis.

Confocal microscopy in proliferating patient cells revealed slightly elongated mitochondria and increased branching, while the size and distribution of nucleoids were normal compared to controls (Figure WS4). Similarly, nucleoids also appeared normal in fixed non‐dividing patient cells; length and branching of mitochondria were not quantitatively assessed (Supplemental Figure S5). Therefore, this suggests that altered mitochondrial protein synthesis in *GMPR* patient cells does not reflect a defect of mtDNA organisation or qualitative changes of the mtDNA sequence.

### 3.10 Mitochondrial DNA replication is not perturbed in *GMPR* patient fibroblasts

Since cytosolic and mitochondrial dNTP levels and relative mtDNA copy number were normal in patient fibroblasts, we challenged mtDNA maintenance by performing a mtDNA depletion‐repletion experiment to assess the rate of mtDNA replication in quiescent cells. mtDNA was depleted using the intercalating agent ethidium bromide for 14 days, and the replenishment of mtDNA was followed for a further 14 days. mtDNA copy number was restored to the original level in patient cells as effectively as control cells (Figure 4B), which suggested no impairment of mtDNA replication.

In the absence of overt mtDNA aberrations in patient fibroblasts, we postulated that challenging the patient cells could stress the phenotype and reveal mild defects in mtDNA replication. By increasing the concentration of the GMPR substrate, GMP, this could lead to increased GTP and therefore alter the ratio of GTP/dGTP pools in cells. To do so, we supplemented the cell culture medium with guanosine (Guo), which in the cell is readily converted to GMP, for 14 days of serum starvation. Following supplementation, quantitative real‐time PCR did not show mtDNA depletion in patient cells (Figure 4C). In addition, long‐range PCR did not detect the presence of large‐scale mtDNA rearrangements (Figure 4D).

## 4 DISCUSSION

Here, we demonstrate an apparent heterozygous mutation in *GMPR*, as another important, albeit rare cause of late‐onset PEO. We show that decreased GMPR protein levels in patient skeletal muscle and proliferating and quiescent fibroblasts could be attributed to aberrant splicing and nonsense‐mediated decay of the mutant, leading to very mild alterations in nucleotide homeostasis. Crucially, the c.547G>C variant in this report is novel and conceivably pathogenic.

*GMPR* encodes guanosine monophosphate reductase, an evolutionarily conserved enzyme from humans to bacteria that catalyses the conversion of the ribonucleotide GMP to IMP, a precursor ribonucleotide for the synthesis of purine nucleotides.^38^ GMPR is highly expressed in skeletal and cardiac muscle and kidney tissue,^41^ but heretofore, is not a known mitochondrial protein and has not been associated with human disease. We experimentally confirmed that GMPR is a cytosolic nucleotide metabolism enzyme, similar to thymidine phosphorylase and p53R2, encoded by *TYMP* and *RRM2B*, respectively, which are known mtDNA maintenance disorder loci.

Reduced expressed of GMPR has been previously evident in skeletal muscle from early‐onset, *TK2‐*related mtDNA depletion patients^42^ and multiple mtDNA deletions in muscle (Figure 1C,D) are a pathological hallmark of mtDNA disorders. Therefore, the expectation was that the GMPR variant caused mtDNA disease owing to perturbed dNTP pools. However, detailed molecular studies suggest that the underlying disease mechanism could be different from any of the well‐established mtDNA disorders. The mitochondrial dNTP pools of GMPR mutant cells are normal and mtDNA copy number is maintained in tissues and proliferating cells. Nor does exit from the cell cycle induce mtDNA depletion, as it does in many other cases and mtDNA replication is not impeded even when the demand is high (Figure 4B). In general, mtDNA copy number is directly proportional to the level of TFAM, as observed in the TFAM‐knockout mouse model.^43^ However, in GMPR patient skeletal muscle, this is not the case, therefore providing an additional mtDNA abnormality. What substitutes TFAM is not known but the change is strongly suggestive of some change in its substrate, that is, the mtDNA sequence or its topology. Moreover, the alternative mtDNA packaging arrangement could be the cause of the accumulated mtDNA deletions and could account for the modest decrease in its expression (Supplemental Figure S2B).

Notwithstanding all of the above, decreased levels of the mitochondrial pyrimidine transporter PNC1 in patient skeletal muscle suggests that there is a nucleotide homeostasis problem. One reason pyrimidine uptake might need to be reduced when the underlying problem relates to purine biosynthesis, would be to maintain an appropriate balance among the dNTP pools, to preserve mtDNA integrity.^36^, ^44^

Finally, we considered whether the depletion of GMPR protein levels may alter the ratio of GTP to dGTP in *GMPR* patient tissue and cells due to increased levels of the GMPR substrate, GMP. Recent evidence suggests that altered rNTP:dNTP ratio leads to aberrant ribonucleotide incorporation in mtDNA that can, in turn, cause stalling of DNA replication in mitochondria.^45^, ^46^ Cultured cell lines from patients with autosomal recessive *MPV17*, *DGUOK* or *TK2* defects that presented reduced levels of mitochondrial dGTP (*MPV17*, *DGUOK*) or dCTP (*TK2*) revealed increased incorporation of rGMP or rCMP in mtDNA.^46^ Aberrant ribonucleotide incorporation into mtDNA can underlie the pathogenesis of multiple mtDNA deletions, as shown in brain tissue from *Mpv17*‐knockout mice, where increased levels of rGMP incorporation in mtDNA was associated with multiple mtDNA deletions.^45^ Thus, tissue specific changes to the ratio of rNTP:dNTPs could explain the presence of mtDNA depletion or multiple deletions in mtDNA maintenance disorders. Decreased GMPR protein or loss of activity in skeletal muscle could increase incorporation of rGMP in mtDNA to deleterious levels, stalling replication and causing multiple mtDNA deletions, but with normal dGTP levels; similar to brain tissue from *Mpv17*‐knockout mice.^45^ However, rNTP:dNTP ratios in cell lines or post‐mitotic tissue derived from patients with dominantly inherited variants causing late‐onset mtDNA maintenance disorders have not been investigated to date.

Overall, our functional data corroborate the subtle clinical phenotype and support our assertion that *GMPR* now represents another important nuclear‐encoded gene associated with PEO and multiple mtDNA deletions; the finding and characterisation of other patients will strengthen this association.

## CONFLICT OF INTEREST

Nothing to declare.

## Supporting information


**Data S1**. Supporting Information.

**Figure S1.** Characterisation of the novel c.547G>C *GMPR* variant on splicing in patient and control fibroblasts. A, Amplification of control and patient fibroblast‐derived cDNA across *GMPR* exons 3‐7, and B, sequencing chromatograms showing wild‐type PCR products from control and patient emetine‐treated fibroblasts. To inhibit nonsense‐mediated decay, control and patient fibroblasts were treated with 100 μg ml^−1^ emetine for 10 hours. C, control; P, patient; NT,‐no template.

**Figure S2.** Analysis of OXPHOS in *GMPR* patient skeletal muscle and proliferating and quiescent fibroblasts. A, Steady‐state levels of OXPHOS subunits in *GMPR* patient and control skeletal muscle homogenates. Antibodies against NDUFB8 (CI), SDHA (CII), UQCRC2 (CIII), MT‐COI (CIV), MT‐COII (CIV) and ATP5B (CV) were used, with SDHA as a loading control. B, Steady‐state levels of GMPR and OXPHOS subunits in *GMPR* patient and control proliferating and quiescent cells. Antibodies against NDUFB8 (CI), UQCRC2 (CIII), MT‐COII (CIV) and ATP5A (CV) were used, with VCL as a loading control. C, ^35^S‐methionine labelling of nascent mitochondrial‐encoded OXPHOS subunits in *GMPR* patient and control proliferating and quiescent cells.

**Figure S3.** in vitro assessment of the Gly183 residue on wild‐type and mutant human GMPR2 substrate binding and activity. A, Structure of GMP/IMP binding site in hGMPR2. Chain A from the structure of the E•IMP•NADPH structure is shown (PDB 2c6q). Residues within 3 Å of IMP plus Gly183 are shown. *h*GMPR2 is shown in salmon, IMP is magenta, residue Gly183 is green. Hydrogen bonds are shown in cyan. B, Activity of GMPR2 and GMPR2‐p.Gly183Arg. NADPH consumption was measured by changes in absorbance at 340 nm at 25°C. Reactions were performed with 100 nM enzyme in 150 μM GMP, 150 μM NADPH, 75 mM Tris‐HCl, pH 7.8, 100 mM KCl, 1 mM EDTA, and 1 mM DTT. *h*GMPR2 (squares) and *h*GMPR2‐p.Gly183Arg (open circles).

**Figure S4.** Analysis of mitochondrial networks and nucleoid morphology in proliferating patient and control fibroblasts. A, The top panel shows representative images of TMRM staining of mitochondrial networks in two controls (C1, C2) and patient proliferating fibroblasts. The lower panel shows representative images of PicoGreen staining of nucleoids in two controls (C1, C2) and patient proliferating fibroblasts. Scale bar = 10 μM. B, Quantitative analysis of (a) aspect ratio and (b) form factor in patient proliferating fibroblasts compared with two controls. Data are represented as the mean ± SEM (n = 10). Two‐tailed unpaired Student's *t*‐test was performed to assess statistical significance.

**Figure S5.** Analysis of mitochondrial networks and nucleoid morphology in non‐dividing patient and control fibroblasts. Confocal images of quiescent control and *GMPR* patient fibroblasts stained for the mitochondrial membrane marker TOM20 (red) and DNA (green).

**Table S1.** Primary antibodies used in this study.

## Data Availability

Data supporting the findings from this study are available from the corresponding author on request.
