# Supplementary material for: Identification of a novel heterozygous guanosine monophosphate reductase (GMPR) variant in a patient with a late‐onset disorder of mitochondrial DNA maintenance
Source: Clin Genet. 2019 Nov 14;97(2):276–86. doi: 10.1111/cge.13652 (PMC7004030; doi:10.1111/cge.13652)
Supplement: Supplementary file 1 — Data S1. Supporting Information. [file CGE-97-276-s001.docx]

SUPPORTING INFORMATION

# Methods

## Whole exome sequencing and candidate variant prioritisation

Called WES variants were restricted to exonic (coding) or splice-site variants with a minor allele frequency (MAF) of less than or equal to 0.01 (1%) from 378 in-house controls and external variant databases including GnomAD (<http://gnomad.broadinstitute.org/>) and the 1000 Genomes Project (<http://www.internationalgenome.org/>). On the basis of no known family history, autosomal dominant (heterozygous) and autosomal recessive (homozygous and compound heterozygous) variants were equally considered. Pathogenic or likely pathogenic variants in known nuclear genes associated with mtDNA maintenance disorders were first excluded, including all 18 genes associated with PEO.. Next, variants were filtered using Gene Ontology (GO)-Terms to prioritise nuclear genes encoding mitochondrial-localised proteins or proteins required for DNA repair or replication. GO-Terms used were the wildcard term ‘mitochondr*’, ‘DNA repair’, ‘replication’, ‘transcription’, ‘nucleotide’, ‘purine’, ‘pyrimidine’, ‘exonuclease’, ‘polymerase’, ‘topoisomerase’, ‘ligase’, ‘helicase’ and ‘nucleoside’. Copy number variants (CNVs) were also examined using the same GO-Terms. Pathogenicity of candidate variants was assessed using *in silico* tools Polyphen-2 (<http://genetics.bwh.harvard.edu/pph2/>), Align GVGD (<http://agvgd.hci.utah.edu/agvgd_input.php>) and SIFT (<http://sift.jcvi.org/>).

The identified *GMPR* variant was confirmed by Sanger sequencing of PCR products amplified using custom forward (5’-TTCTCATGCCAACAGCTCAC-3’) and reverse (5’-ACTTTTCATCAGGGGCTTCC-3’) primers flanked with universal M13-derived tags, using the BigDye Terminator v3.1 Cycle Sequencing kit on the ABI 3130*xl* Genetic Analyser, according to manufacturer’s guidelines.

*cDNA studies in skeletal muscle and cultured fibroblasts*

Total RNA was extracted from patient and age-matched control skeletal muscle using the ReliaPrep RNA Tissue MiniPrep system (Promega). Total RNA was extracted from patient and control cultured fibroblasts using the ReliaPrep RNA Cell MiniPrep system (Promega). Cultured fibroblast medium was supplemented with 100μg ml^-1^ emetine for 10h to inhibit nonsense-mediated decay. RNA was reverse transcribed to cDNA using the GoScript Reverse Transcription system (Promega). Custom forward (5’-AGCCGAGGTGGATCTTGAA-3’) and reverse (5’-AGTCCCCCGAGAATATCCAG-3’) primers flanked with universal M13-derived tags were used to amplify *GMPR* exons 3 to 7. PCR products were separated by gel electrophoresis and Sanger sequenced as described above.

*Cloning of hGMPR2 gene and mutagenesis*

A cDNA clone (pOTB7) encoding full length *h*GMPR2 (MGC; accession no. BC008021) was purchased from Thermo Scientific (MHS1011-62195). Phusion Hot Start DNA polymerase was used for cloning procedure. The coding sequence was amplified from cDNA for insertion into pET28a. The 5` of each primer correspond to the pET28a vector and 3` ends correspond to the *h*GMPR2 sequence (forward primer = TGGTGCCTCGTGGTAGCCATATGCCTCATATTGACAACGATGT; reverse primer = CTCAGCTTCCTTTCGGGCTTTGTTACTAGCACGCCTCACTGAAGATTG). The resulting product was then used in a final PCR with empty pET28a vector to afford pET28a-*h*GMPR2, which expresses *h*GMPR2 with an N-terminal tag containing a hexa-His sequence with a thrombin cleavage site (MGSSHHHHHHSSGLVPRGSH). The procedure was repeated to create *h*GMPR2-G183R with an additional PCR to insert the mutation (G183R forward = CTCTTCACTGCTGTCCATAAGCA and G183R reverse = TTTTCTTCCGAGTAGTACACACAGAGCGTGGCCCAATTCCCACTTT). DNA sequencing of the plasmids confirmed the desired sequence.

*Overexpression and purification of hGMPR2 and hGMPR2-G183R*

The expression and purification of recombinant *h*GMPR2 and the p.Gly183Arg mutant was carried out as described previously with small modifications.^1^ Briefly, the crude cell lysate from a 2-litre culture was loaded onto Ni-NTA agarose resin (Molecular Cloning Laboratories Inc.) and incubated overnight. The resin was washed sequentially with 5mM, 30mM and 60mM imidazole in buffer 1 (100mM potassium phosphate, pH 7.8, 500mM KCl, 1mM BME, 10% glycerol). *h*GMPR2 was eluted in 250mM imidazole in buffer 1. The fractions with high enzyme activity were collected and dialyzed against buffer 2 (75mM Tris-HCl, pH 7.8, 100mM KCl, 1mM DTT, 0.5mM EDTA, 5% glycerol). Protein purity was verified by SDS-PAGE. The protein concentration was determined by A_280_ using the extinction coefficient calculated with ProtParam.^2^

*Enzyme kinetics*

Standard GMPR assays were conducted for GMPR2 at 25°C in 75mM Tris-HCl, pH 7.8, 100mM KCl, 1mM EDTA, and 1mM DTT (GMPR buffer) with varying GMP and NADPH by monitoring the disappearance of NADPH spectrophotometrically at 340nm. NADPH consumption was measured for *h*GMPR2-p.Gly183Arg GMPR2 at GMP (150 μM) and NADPH (150 μM) at 25°C.

## Cellular and sub-mitochondrial fractionation

Cells from two 300 cm^2^ flasks were harvested at approximately 80% confluency, resuspended in H-buffer (0.6M Mannitol, 10mM Tris-HCl [pH 7.4], 1mM EGTA, freshly supplemented with 0.1% BSA and 1mM PMSF), and homogenised (glass Teflon homogeniser, 15 passes). Cytosolic fraction (post-mito spn) and mitochondrial pellet were obtained following standard differential centrifugations. Mitoplasts were prepared by resuspending mitochondria in hypotonic buffer (10mM Tris-HCl [pH 7.4]) and treated with Proteinase K (1ug mg^-1^ of mitochondria) in H-buffer; digestion was inhibited by addition of 5mM PMSF. To prepare the inner mitochondrial membrane (IMM) protein fraction, Proteinase K-treated mitoplasts from HeLa cells were pelleted, dissolved in 100mM Na_2_CO_3_ and incubated on ice, followed by ultracentrifugation. Final pellet was directly resuspended in 1X Laemmli buffer (2% SDS, 10% glycerol, 5% 2-mercaptoethanol, 0.002% bromphenol blue and 62.5mM Tris HCl [pH 6.8]).

## Confocal Microscopy

Control and patient proliferating fibroblasts were grown in glass bottom imaging dishes (iBidi) to assess mitochondrial networks and nucleoid morphology, as described previously.^3^ Proliferating fibroblasts were incubated in 5 nM tetramethylrhodamine methyl ester (TMRM) (Invitrogen) for 45 min, followed by 3μl ml-1 PicoGreen solution (Life Technologies). Images were captured (n=10 each) using the Nikon A1R Inverted point scanning confocal microscope with a 63x oil immersion objective, in an enclosed environment chamber at 37°C with 5% CO2 using resonant scanning for scanning and an increased frame rate. Z-stacks were processed and analysed using ImageJ. Images were binarised to allow automated quantification of mitochondrial morphological features; aspect ratio (AR) reflected the length, perimeter and area of mitochondria. Form factor (FF) reflected the length and degree of branching, which was calculated by perimeter2/4π*area. The average AR and FF between the patient cell line and each control was compared and subjected to two-tailed unpaired student’s t-test.

For quiescent fibroblasts, cells were seeded into microscopy chambers. When confluence was reached, serum starvation was started by changing the medium from 10% to 0.1% FBS. Medium was changed every 3 days and cells were fixed 14 days after starvation was started. Anti-DNA and TOM20 immunostaining was performed as described previously,^4^ using anti-DNA (Progen, 1:200) and anti-Tom20 (Santa Cruz, 1:500) as primary antibodies.

## Nucleoid organisation in skeletal muscle

Fresh frozen muscle sections of 10 µm thickness were fixed with 4% PFA for 10 minutes, washed with PBS three times (5 min each), and permeabilised in PBS, 0.3% Triton-X for 45 min at RT. After three washes in PBS, 0.2% Tween (PBS-T) slides were blocked in PBS-T, 5% goat serum for 2 h and subsequently incubated with anti-TOM20 (Abcam, 1:200) and anti-DNA (Progen, 1:200) antibodies in PBS-T, 5 % goat serum at 4 ̊C overnight. After three washes secondary antibodies (AlexaFluor® 488 Anti-Mouse IgG, IgM and AlexaFluor® 568 Anti-Rabbit IgG, both Thermo Fisher Scientific) were incubated in PBS-T for 2 h at RT. Slides were mounted using ProLong® Gold Antifade Reagent with DAPI (Thermo Fisher Scientific) and imaged on a Nikon Ti Inverted Confocal Microscope with a 60X immersion objective.

# Results

## In vitro studies

The Gly183 residue is close to the active site,^5^ adjacent to a residue that makes a hydrogen bond to the phosphate of the product IMP (**Supplemental Figure 3A**). This observation suggests that substitution of glycine with arginine will perturb substrate binding and decrease enzymatic activity. Due to availability and the homology between human GMPR and GMPR2, we expressed and characterised wild-type human GMPR2 and the p.Gly183Arg variant (*h*GMPR2-G183R) *in vitro*. The steady-state kinetic parameters for wild-type GMPR2 were *K*_m_(GMP) = 3.3 ± 1.4 mM, *K*_m_(NADPH) = 15 ± 3 mM and *V*_max_ = 0.10 ± 0.01 s^-1^. The GMPR2-G183R mutant decreased enzymatic activity by a factor of 80 (GMPR2 v/[E] = 0.091 ± 0.007 s^-1^; GMPR2-G183R, v/[E]= 0.001±0.001 at 150 μM GMP and 150 μM NADPH) (**Supplemental Figure 3B**). This activity was too low to determine the values of *K*_m_ and *V*_max_ for GMPR2-G183R, though it is apparent that the values of *K*_m_ for both substrates >> 150 μM. Therefore, our *in vitro* data confirm that the Gly183 residue is critical for enzymatic activity.

# Supplemental Figure Legends

**Supplemental Figure 1 Characterisation of the novel c.547G>C *GMPR* variant on splicing in patient and control fibroblasts.** (**A**) Amplification of control and patient fibroblast-derived cDNA across *GMPR* exons 3-7 and (**B**) sequencing chromatograms showing wild-type PCR products from control and patient emetine-treated fibroblasts. To inhibit nonsense-mediated decay, control and patient fibroblasts were treated with 100μg ml^-1^ emetine for 10h. C – control; P – patient; NT – no template.

**Supplemental Figure 2** **Analysis of OXPHOS in *GMPR* patient skeletal muscle and proliferating and quiescent fibroblasts.** (**A**) Steady-state levels of OXPHOS subunits in *GMPR* patient and control skeletal muscle homogenates. Antibodies against NDUFB8 (CI), SDHA (CII), UQCRC2 (CIII), MT-COI (CIV), MT-COII (CIV) and ATP5B (CV) were used, with SDHA as a loading control. (**B**) Steady-state levels of GMPR and OXPHOS subunits in *GMPR* patient and control proliferating and quiescent cells. Antibodies against NDUFB8 (CI), UQCRC2 (CIII), MT-COII (CIV) and ATP5A (CV) were used, with VCL as a loading control. (**C**) ^35^S-methionine labelling of nascent mitochondrial-encoded OXPHOS subunits in *GMPR* patient and control proliferating and quiescent cells.

**Supplemental Figure 3 *In vitro* assessment of the Gly183 residue on wild-type and mutant human GMPR2 substrate binding and activity.** (**A**) Structure of GMP/IMP binding site in hGMPR2. Chain A from the structure of the E•IMP•NADPH structure is shown (PDB 2c6q). Residues within 3 Å of IMP plus Gly183 are shown. *h*GMPR2 is shown in salmon, IMP is magenta, residue Gly183 is green. Hydrogen bonds are shown in cyan. (**B**) Activity of GMPR2 and GMPR2-p.Gly183Arg. NADPH consumption was measured by changes in absorbance at 340 nm at 25 °C. Reactions were performed with 100nM enzyme in 150 μM GMP, 150 μM NADPH, 75 mM Tris-HCl, pH 7.8, 100 mM KCl, 1 mM EDTA, and 1 mM DTT. *h*GMPR2 (squares) and *h*GMPR2-p.Gly183Arg (open circles).

**Supplemental Figure 4** **Analysis of mitochondrial networks and nucleoid morphology in proliferating patient and control fibroblasts.** (**A**) The top panel shows representative images of TMRM staining of mitochondrial networks in two controls (C1, C2) and patient proliferating fibroblasts. The lower panel shows representative image of PicoGreen staining of nucleoids in two controls (C1, C2) and patient proliferating fibroblasts. Scale bar = 10μM. (**B**) Quantitative analysis of (i) aspect ratio and (ii) form factor in patient proliferating fibroblasts compared with two controls. Data are represented as the mean ± SEM (n=10). Two-tailed unpaired student’s t-test was performed to assess statistical significance.

**Supplemental Figure 5** **Analysis of mitochondrial networks and nucleoid morphology in non-dividing patient and control fibroblasts.** Confocal images of quiescent control and *GMPR* patient fibroblasts stained for the mitochondrial membrane marker TOM20 (red) and DNA (green).

# References

1. Rosenberg MM, Redfield AG, Roberts MF, Hedstrom L. Substrate and cofactor dynamics on guanosine monophosphate reductase probed by high resolution field cycling31 P NMR relaxometry. *J Biol Chem*. 2016;291(44):22988-22998. doi:10.1074/jbc.M116.739516

2. Gasteiger E, Hoogland C, Gattiker A, et al. Protein Identification and Analysis Tools on the ExPASy Server. In: *The Proteomics Protocols Handbook*. ; 2005:571-607. doi:10.1385/1592598900

3. Oláhová M, Thompson K, Hardy SA, et al. Pathogenic variants in HTRA2 cause an early-onset mitochondrial syndrome associated with 3-methylglutaconic aciduria. *J Inherit Metab Dis*. 2017;40(1):121-130. doi:10.1007/s10545-016-9977-2

4. Dalla Rosa I, Durigon R, Pearce SF, et al. MPV17L2 is required for ribosome assembly in mitochondria. *Nucleic Acids Res*. 2014. doi:10.1093/nar/gku513

5. Li J, Wei Z, Zheng M, et al. Crystal structure of human guanosine monophosphate reductase 2 (GMPR2) in complex with GMP. *J Mol Biol*. 2006;355(5):980-988. doi:10.1016/j.jmb.2005.11.047
