# Supplementary figures and images for: Identification of a novel heterozygous guanosine monophosphate reductase (GMPR) variant in a patient with a late‐onset disorder of mitochondrial DNA maintenance
Source: Clin Genet. 2019 Nov 14;97(2):276–86. doi: 10.1111/cge.13652 (PMC7004030; doi:10.1111/cge.13652)

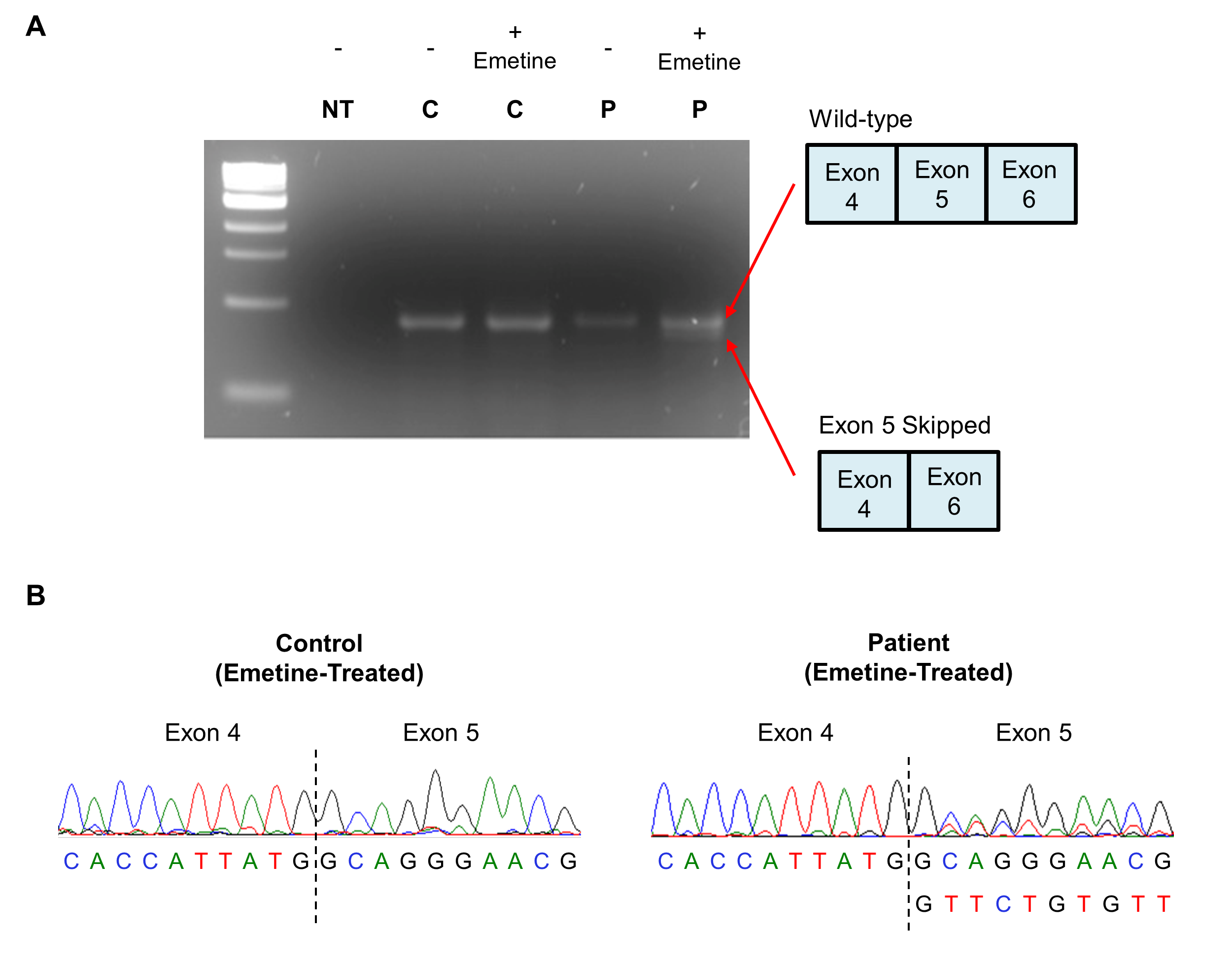

Supplement: Supplementary file 2 — Figure S1. Characterisation of the novel c.547G>C GMPR variant on splicing in patient and control fibroblasts. A, Amplification of control and patient fibroblast‐derived cDNA across GMPR exons 3‐7, and B, sequencing chromatograms showing wild‐type PCR products from control and patient emetine‐treated fibroblasts. To inhibit nonsense‐mediated decay, control and patient fibroblasts were treated with 100 μg ml−1 emetine for 10 hours. C, control; P, patient; NT,‐no template. [file CGE-97-276-s002.tif]

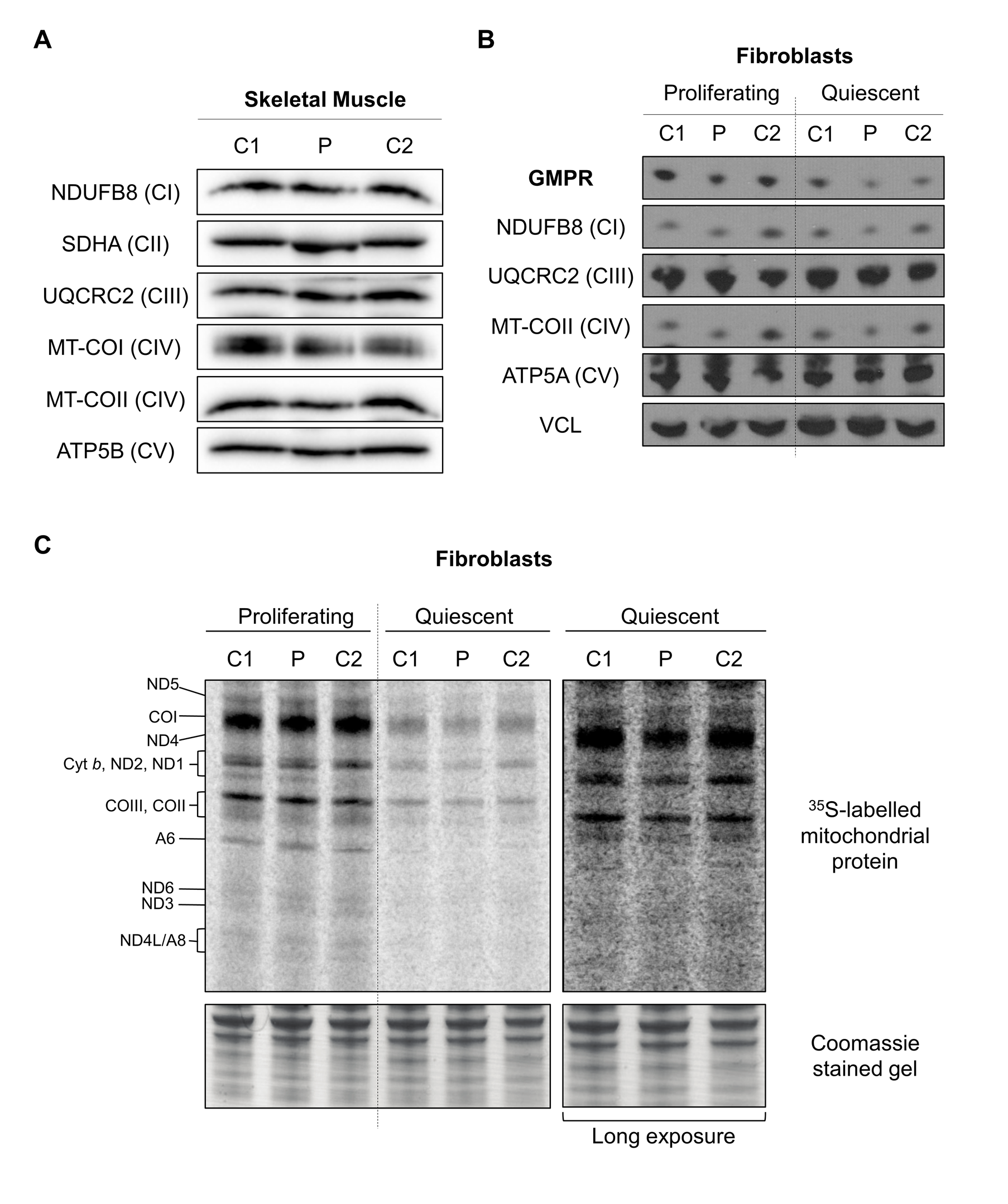

Supplement: Supplementary file 3 — Figure S2. Analysis of OXPHOS in GMPR patient skeletal muscle and proliferating and quiescent fibroblasts. A, Steady‐state levels of OXPHOS subunits in GMPR patient and control skeletal muscle homogenates. Antibodies against NDUFB8 (CI), SDHA (CII), UQCRC2 (CIII), MT‐COI (CIV), MT‐COII (CIV) and ATP5B (CV) were used, with SDHA as a loading control. B, Steady‐state levels of GMPR and OXPHOS subunits in GMPR patient and control proliferating and quiescent cells. Antibodies against NDUFB8 (CI), UQCRC2 (CIII), MT‐COII (CIV) and ATP5A (CV) were used, with VCL as a loading control. C, 35S‐methionine labelling of nascent mitochondrial‐encoded OXPHOS subunits in GMPR patient and control proliferating and quiescent cells. [file CGE-97-276-s003.tif]

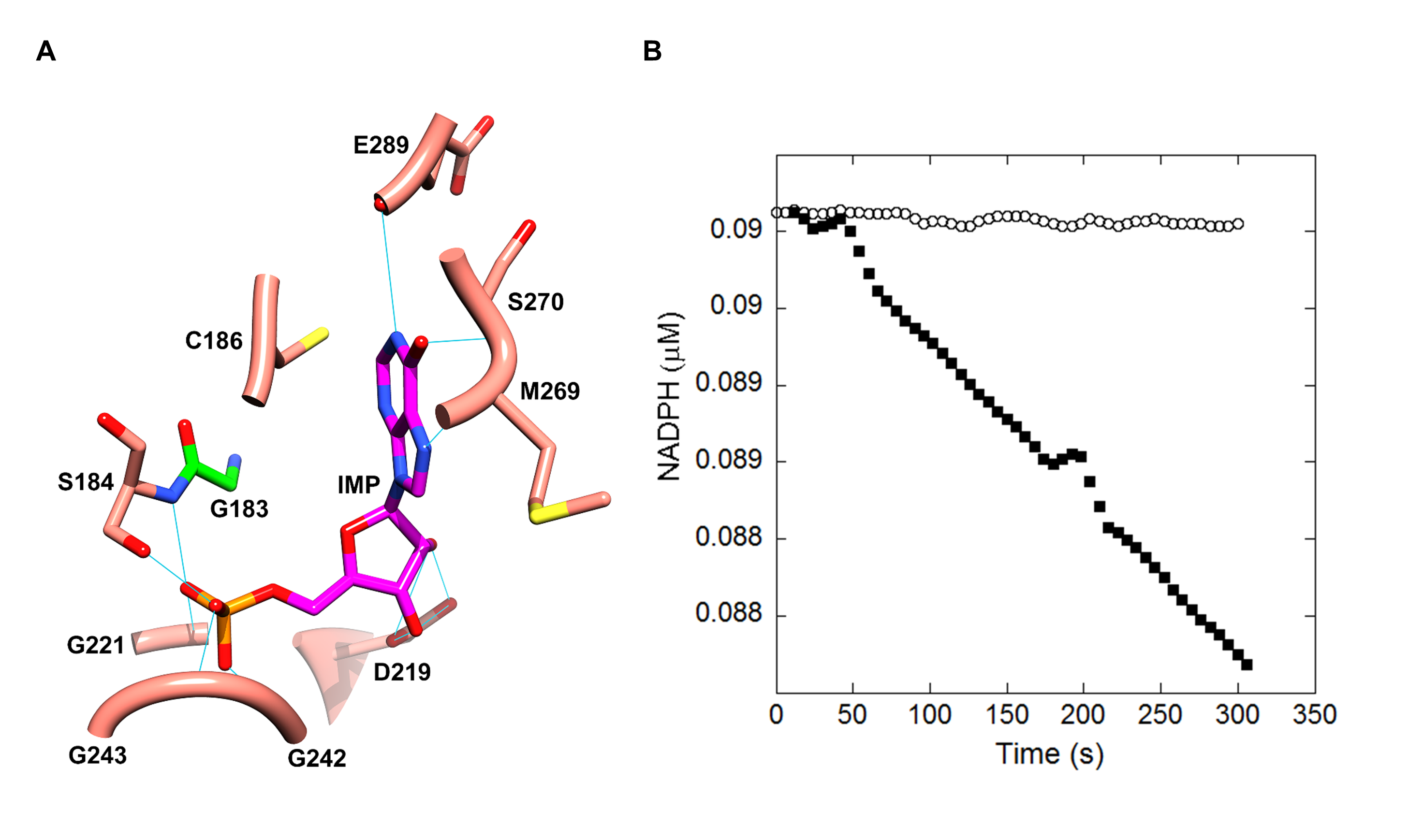

Supplement: Supplementary file 4 — Figure S3. in vitro assessment of the Gly183 residue on wild‐type and mutant human GMPR2 substrate binding and activity. A, Structure of GMP/IMP binding site in hGMPR2. Chain A from the structure of the E•IMP•NADPH structure is shown (PDB 2c6q). Residues within 3 Å of IMP plus Gly183 are shown. hGMPR2 is shown in salmon, IMP is magenta, residue Gly183 is green. Hydrogen bonds are shown in cyan. B, Activity of GMPR2 and GMPR2‐p.Gly183Arg. NADPH consumption was measured by changes in absorbance at 340 nm at 25°C. Reactions were performed with 100 nM enzyme in 150 μM GMP, 150 μM NADPH, 75 mM Tris‐HCl, pH 7.8, 100 mM KCl, 1 mM EDTA, and 1 mM DTT. hGMPR2 (squares) and hGMPR2‐p.Gly183Arg (open circles). [file CGE-97-276-s004.tif]

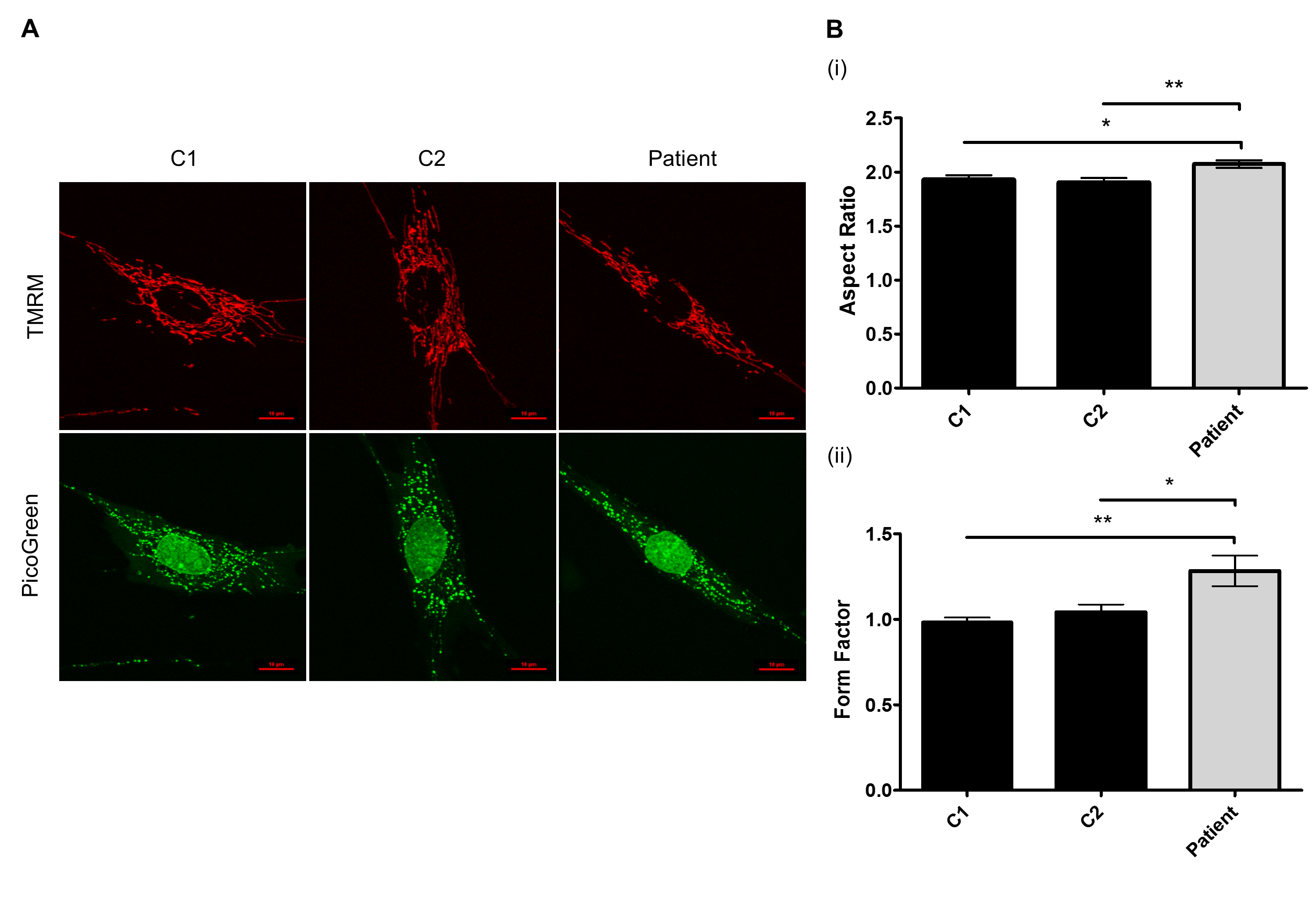

Supplement: Supplementary file 5 — Figure S4. Analysis of mitochondrial networks and nucleoid morphology in proliferating patient and control fibroblasts. A, The top panel shows representative images of TMRM staining of mitochondrial networks in two controls (C1, C2) and patient proliferating fibroblasts. The lower panel shows representative images of PicoGreen staining of nucleoids in two controls (C1, C2) and patient proliferating fibroblasts. Scale bar = 10 μM. B, Quantitative analysis of (a) aspect ratio and (b) form factor in patient proliferating fibroblasts compared with two controls. Data are represented as the mean ± SEM (n = 10). Two‐tailed unpaired Student's t‐test was performed to assess statistical significance. [file CGE-97-276-s005.tif]

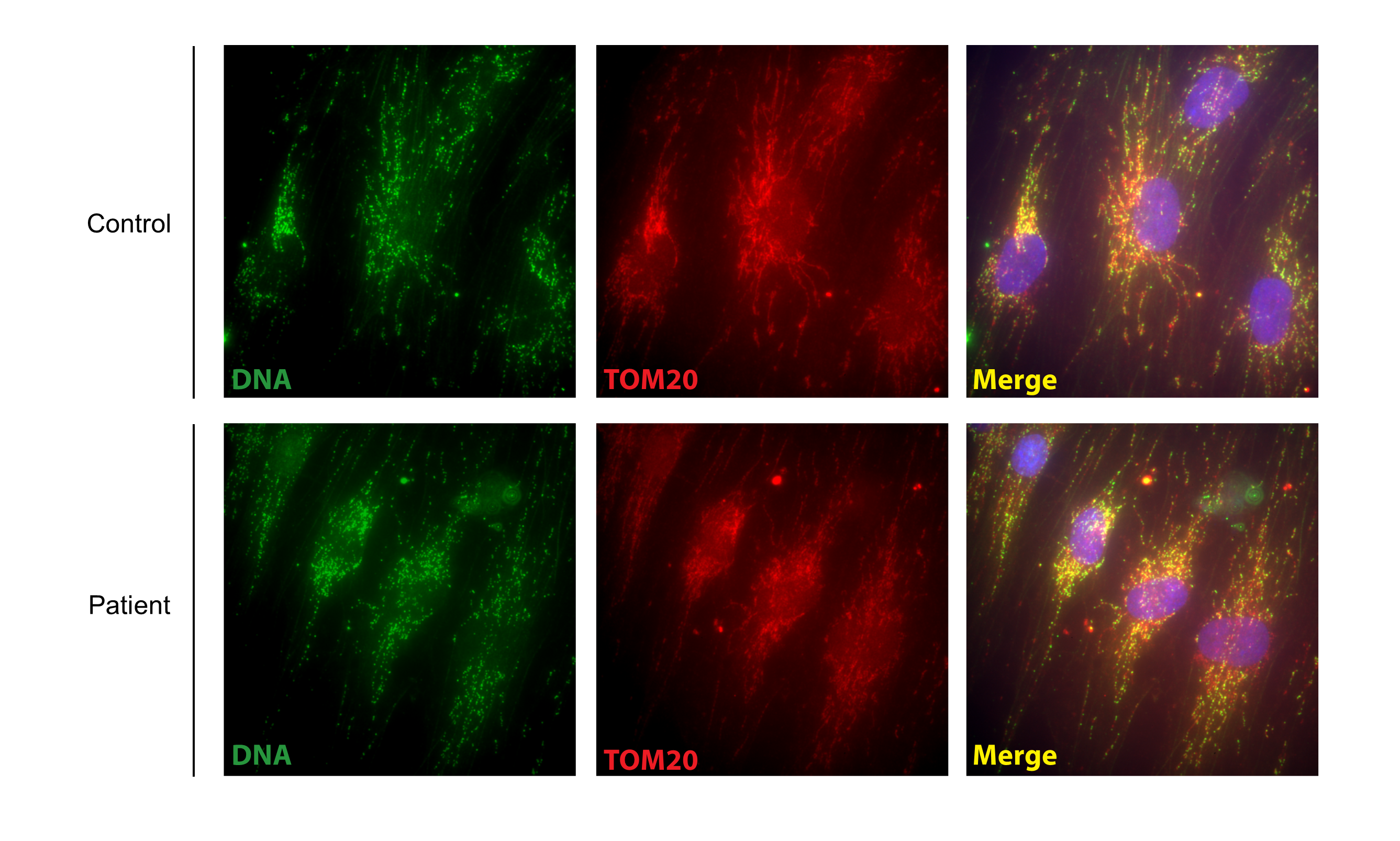

Supplement: Supplementary file 6 — Figure S5. Analysis of mitochondrial networks and nucleoid morphology in non‐dividing patient and control fibroblasts. Confocal images of quiescent control and GMPR patient fibroblasts stained for the mitochondrial membrane marker TOM20 (red) and DNA (green). [file CGE-97-276-s006.tif]
