# Supplementary material for: Identification of a novel heterozygous guanosine monophosphate reductase (GMPR) variant in a patient with a late‐onset disorder of mitochondrial DNA maintenance
Source: Clin Genet. 2019 Nov 14;97(2):276–86. doi: 10.1111/cge.13652 (PMC7004030; doi:10.1111/cge.13652)
Supplement: Supplementary file 7 — Table S1. Primary antibodies used in this study. [file CGE-97-276-s007.docx]

# Supplemental Table 1. Primary antibodies used in this study.

| Antibody | Company | Catalogue Number |
| --- | --- | --- |
| GMPR | Abcam Sigma Aldrich | ab118752 SAB1101144 |
| NDUFB8 | Abcam | ab110242 |
| SDHA | Abcam | ab14715 |
| SDHB | Abcam | ab14714 |
| UQCRC2 | Abcam | ab14745 |
| MT-COI | Abcam | ab14705 |
| MT-COII | Abcam | ab110258 |
| ATP5A | Abcam | ab14748 |
| ATP5B | Abcam | ab14730 |
| TFAM | Abcam | ab119684 |
| LONP1 | Sigma Aldrich | HPA002192 |
| R1 | Santa Cruz | sc-22786 |
| p53R2 | Abcam | ab8105 |
| PNC1 | Abcam | ab97820 |
| AAC | Gift from Dr Edmund Kunji, antibody produced in chicken. | |
| POLG | Santa Cruz | sc-5930 |
| GAPDH | Abcam | ab8245 |
| VCL | Abcam | ab18058 |
| α-tubulin | Abcam | ab7291 |

# 
